# *In vitro* toxicity assessment of uranium particulates on different human lung epithelial cell models

**DOI:** 10.1371/journal.pone.0334247

**Published:** 2025-10-31

**Authors:** Shepard C. Moore, Laura M. Lilley, Julie Strickland, Mohammad Omar Ishak, William Conor Emberley, Brian L. Scott, Gregory L. Wagner, Warren J. Oldham, Murray E. Moore, Harshini Mukundan, Jennifer Foster Harris

**Affiliations:** Los Alamos National Laboratory, Los Alamos, New Mexico, United States of America; Ural Federal University named after the first President of Russia B N Yeltsin Institute of Physics and Technology: Ural’skij federal’nyj universitet imeni pervogo Prezidenta Rossii B N El'cina Fiziko-tehnologiceskij institut, RUSSIAN FEDERATION

## Abstract

Inhalation of uranium aerosols produced via human activities such as mining can pose a threat to human respiratory systems. Uranium oxide particulates emit short-range alpha particles that elicit DNA and direct damage, beyond associated physiochemical heavy-metal toxicity, to internal epithelial tissues. The availability of reliable *in vitro* models to study radiation exposure can greatly enhance our ability to understand and combat the biological impacts of exposure. However, the toxicological effects of alpha emissions and/or the oxidation states of uranium particulates vary across different human lung epithelial cell models and have not been systematically compared. We have endeavored to address this limitation by comparing impacts in three different human lung cell models: primary human bronchial and tracheal epithelial cells, primary human small airway epithelial cells, and human adenocarcinoma alveolar basal epithelial cells. Other studies have mainly investigated the toxicity of depleted uranium. Here, we compared the exposure of uranium oxide particulates (U_3_O_8_ and UO_3_) of different enrichment states on the chosen cell systems. Each cell model was exposed to 0.1, 1, 10, 50, 100, and 500 µg/mL of depleted U_3_O_8_, highly-enriched U_3_O_8_, and natural UO_3_ particulates for 24 hours in submerged monolayer cultures. We compared viability and superoxide dismutase activity results across cell lines and uranium enrichment/ oxidative states. The results showed that 1) the oxide state of the particulates affected cell viability, implying that uranium’s different oxidation states contribute to different toxicological responses, and 2) each cell model reacts differently when exposed to uranium oxides, which may provide insights into the mechanistic processes associated with the exposure of radiological particulates on different biological systems. For instance, increased uranium enrichment corresponds to increased toxicity for the primary cells, but not for the immortalized cells. Our study shows that a holistic approach that incorporates similarities between model systems and types of radionuclides is required to truly develop empirical solutions for radiation exposure.

## Introduction

Acute and chronic exposure to inhaled alpha (α)-emitting radioactive particulates, like uranium (U), from environmental, occupational, and enriched sources may lead to health risks to the human respiratory system due to its chemical and radiological toxicity [1–5]. Studies indicate that the epithelial lining of the lungs is the most susceptible area to internalized ionizing radiation, especially α-emissions [6]. Alpha particles cause cellular damage by directly breaking DNA strands, disrupting cell homeostasis, destroying cell membranes; and by indirectly generating reactive oxygen/nitrogen species to ravage cell integrity [7–15].

Uranium is a common heavy metal found in the earth’s crust. There are three naturally occurring isotopes U-238 (*t*_1/2 _= 4.46x10^9^ y), U-235 (*t*_1/2 _= 7.04x10^8^ y), and U-234 (*t*_1/2 _= 2.46x10^5^ y) all producing α-particles [16]. Uranium is extracted from mines, used as fuel in nuclear power plants, found in groundwater via soil, and is a parent of many radioisotopes for medical, industrial, and military defense purposes [17]. Uranium has a tunable isotopic ratio, meaning that it is possible to vary the amount of general radioactivity—for the work in this manuscript, specifically α-emissions—in each sample to depleted (DU), natural (NatU), or highly enriched (HEU) values by decreasing or increasing the amount of its most fissile isotope, U-235 [18]. DU is a byproduct of the production of HEU and is used for many military munitions, radiation shielding, nuclear power plants, and nuclear weapons [19]. NatU, derived from uranium mining, can be deposited in soil, rock, and water, which can consequently leach into human infrastructure. One of the primary properties of NatU is its potential to be enriched to HEU or DU [20]. HEU is typically used for nuclear power, nuclear weapons, naval propulsion, and research reactors [21,22]. Whereas each of these uranium enrichment states can elicit negative health effects in human lung epithelial cells, the correlation between cellular toxicity and enrichment remains poorly understood [23].

The oxidation state of the uranium can vary, influencing associated biological effects on living systems. The common oxidation state(s) of uranium are at U(IV) and U(VI), for example, uranium dioxide (UO_2_) and uranium trioxide (UO_3_), respectively [24]. A mixed valence configuration is possible for triuranium octoxide (U_3_O_8_) that has potential combinations of U(IV), U(V), and U(VI) states [25]. The different oxidative states vary with regard to thermostability and reactivity as well. Uranium is usually shipped between facilities in its thermodynamically stable state, U_3_O_8_, and not as UO_2_, which is relatively less stable and can undergo further oxidation in the presence of air or aqueous buffers to UO_3_ or U_3_O_8_ [26].

The form of the isotope used in most toxicity studies exploring biological impact is dissolved uranium (e.g., uranium salts such as uranyl acetate) [27,28]. However, in one study, Harris et al. showed that 60% of inhaled uranium particulates were still present in the human body after 32 days of exposure, highlighting the urgent need to study insoluble uranium particulate species in a biological context [29]. U_3_O_8_ and UO_3_ particulates have poor solubilities in water and are known to be retained within the lungs for several years, allowing for long-term exposure to the heavy metal and high-energy α-emissions [30–32]. Differences in sensitivity of upper or lower airways to radioactive particulates remain poorly studied [33,34]. *In vitro* toxicological (human) cell models provide a potential path to assess uranium particulate exposure effects [35]. However, the physiological relevance of these studies, translational value of the garnered information, and the variability in outcomes across various cell models remain poorly characterized and should be further explored.

Adenocarcinoma alveolar basal epithelial cells (A549), a human alveolar type II pulmonary epithelial cell, are often used as a lung surrogate due to their ease of cultivation, proliferation, and (almost) unlimited supply [36,37]. However, A549s are an immortalized cell line that is known to introduce unique artifacts into research results due to their chronic genetic drift [38–40]. Such genetic drift has potentially skewed toxicological interpretations of assumed toxicants due to A549’s lowered sensitivity, as seen in tobacco smoke and lipopolysaccharide (LPS) [41–43]. Primary human lung cells, such as primary human bronchial and tracheal epithelial cells (HBTEC) and primary human small airway epithelial cells (HSAEC), may provide more physiologically relevant *in vitro* models [41]. Unlike immortalized A549s, primary cell lines have limited lifetimes, passage numbers, and unique genomic profiles from each donor, elevating the complexity and cost of experimentation [44]. Thus, an ideal *in vitro* model system for studying the impact of radiation does not exist, and further investigation is needed to characterize the tissues used in understanding the radiobiological effects of α-emitting materials on relevant human-specific respiratory systems [45–47].

The purpose of this study was to begin addressing the aforementioned limitations. We performed simultaneous comparative investigations of cytotoxic and mechanistic effects of increased enrichment and oxidation state of uranium particulates on several *in vitro* human epithelial lung cell models—HBTEC, HSAEC, and A549. We explore differences in upper and lower respiratory airway cells to radioactive particulates via comparison of toxicological response of upper (HBTEC), middle (HSAEC), and lower (A549) respiratory tract cells. We hypothesized that the most cellular cytotoxicity and damage will be evident with HEU, even at lower concentrations, because of significantly increased α-radiation activity. Other investigators have hypothesized that primary cell lines will be more susceptible to exposure because of their less robust nature compared to immortalized cell lines [48]. To evaluate this, we used multi-modal comprehensive analytics to characterize both the particulates and the biological outcomes.

Differences in susceptibility between cell models can inform on potential use cases for each model for future α-emitter radionuclide toxicity studies. Additionally, due to uranium’s lower activity even when highly enriched, our findings may enable identification of a model system that can provide insight into the effects of other, more hazardous, α-emitters or heavy metals in the respiratory system.

## Materials and methods

Uranium oxide particulates were synthesized and characterized to validate U-235 isotopic ratios (DU < 0.7%, NatU ≥ 0.7%, HEU > 20%), and diameters were respirable for humans according to the International Commission on Radiological Protection (ICRP) (<10 µm mass median aerodynamic diameter, MMAD) [49].

### Uranium oxide synthesis

Each uranium oxide sample (DU U_3_O_8_, NatU UO_3_, and HEU U_3_O_8_) was freshly prepared and purified to reduce impurities associated with aging of radiological materials and individually followed the subsequent synthesis procedure. A solid metal bar of uranium (DU 100 g, NatU 4 g, HEU 0.495 g) was dissolved in ~100 mL of Optima grade concentrated hydrochloric acid (HCl, FisherSci, A466-500), with additional hydrogen peroxide (H_2_O_2_, FisherSci, P170-500) to speed up dissolution, in a round- bottom flask, producing uranyl (UO_2_^2+^). The dissolved metal was purified in accordance with Wilkerson et al. using a Reillex HPQ Polymer Ion Exchange column (Vertellus) with concentrated hydrochloric acid (HCl) and 0.01 M HCl as an eluent [50]. Once the purified uranium was collected, ammonium hydroxide (NH_4_OH, FisherSci, A470-500) was added dropwise to reach a desired pH of 3.0, then H_2_O_2_ was added dropwise to produce the precipitate uranyl peroxide hydrate (UO_2_(O_2_)·*x*H_2_O) lowering the pH back to 1.0. The process was repeated by adjusting the pH to 3.0 with NH_4_OH until the addition of H_2_O_2_ did not cause a decrease in pH. The yellow precipitate was then vacuum filtered through a fine glass frit, washed three times with ~30 mL of MilliQ water, and left to air-dry overnight. Once dry, the recovered uranium was crushed to a fine powder using a mortar and pestle, loaded in a platinum foil- lined ceramic boat, and then fired in a tube furnace at 800 °C for 20 hours in air, producing a black U_3_O_8_ powder [51,52]. The olive green natural UO_3_ powder was produced the same way as depleted and highly enriched U_3_O_8_, except the firing time was increased to 40 hours. A diagram of the synthesis is shown in S1 Fig in S1 File.

### Particulate preparation and characterization

Control particulates titanium dioxide (TiO_2_, rutile powder, < 5 µm, > 99.9% trace metals basis, Lot# MKCG2282) and silicon dioxide (SiO_2_, ~ 99%, 0.5 - 10 µm, approximately 80% between 1–5 µm, Lot# SLCH1152) were obtained from Sigma Aldrich. All U_3_O_8_ and UO_3_ particulates were pushed through individual <25 µm sieves (Industrial Netting) with a fine paint brush in a chemical/radiological fume hood to closely achieve a target size of <10 µm MMAD. The sieved material was collected on aluminum foil underneath the sieve and carefully transferred to labeled glass scintillation vials for long-term storage.

#### Powder X-ray diffraction (p-XRD) analysis.

All synthesized U_3_O_8_ and UO_3_ samples were verified using powder X-ray diffraction (p-XRD) analysis (S2 Fig in S1 File) as described previously [50]. Briefly, powder patterns of samples at time 0 were collected on a Bruker D8 Discover diffractometer equipped with either a Hi-Star area detector or a high-resolution NiI scintillation detector and monochromatized Cu Kα X-rays. Qualitative analyses were performed using JADE 9.0 search/matching and the powder diffraction file (PDF-4+) 2013 database from the International Center for Diffraction Data (ICDD). PDF numbers listed in the figures were taken from the ICDD database PDF-4 + . Slight non-stoichiometries in hydration or oxygen content can cause variations between reference lines reported for a given chemical species.

#### Isotopic verification.

The uranium isotopic composition of the three samples (DU U_3_O_8_, NatU UO_3_, HEU U_3_O_8_) was determined using a quadrupole inductively-coupled plasma mass spectrometer (Thermo X-series II ICP-MS equipped with a standard spray chamber). The DU U_3_O_8_ sample was received as a dissolved aliquot in 4 M nitric acid (HNO_3_) at an approximate uranium concentration of 10 µg/mL. This sample was prepared for analysis using 2% HNO_3_ for serial dilution. The NatU UO_3_ and HEU U_3_O_8_ samples (~5 mg) were dissolved in separate operations using 3 M HNO_3_, and then prepared for analysis after serial dilution using 2% HNO_3_. Analytical acids were Optima grade and were prepared as stock reagents using 18.2 MΩ-cm (MilliQ) water.

Instrumental performance and mass bias corrections were determined using an analytical dilution of the Institute for Reference Materials and Measurements (IRMM) 74/1. Results are reported as uranium isotope atom percent composition (Table 1) and as atom ratios relative to U-235 (S1 Table in S1 File), with indicated measurement uncertainty representing 1-sigma standard deviation. Each sample was prepared and analyzed independently alongside an isotopically similar uranium standard to evaluate measurement quality. These data are presented along with the certified, or consensus, reference values for National Bureau of Standards Standard Reference Materials (SRMs) U-005, U-005A, U-960, and U-930 in S2 and S3 Tables in S1 File. The number of α-emissions per 24 hours per 100 µg of each uranium enrichment was calculated by multiplying the specific activity of each isotope at their respective isotopic composition percentage by the 100 µg sample to justify a significant difference of DU/NatU (1.24E + 05 and 2.24E + 05) α-radiation activity compared to our 90% U-235 enriched HEU (2.01E + 07).

**Table 1:** Isotopic analysis (percent) and estimation of α-emission rate of synthesized uranium oxide particulates. Uranium oxide samples were isotopically analyzed using a quadrupole inductively-coupled plasma mass spectrometer and compared to isotopically similar standardized uranium material. The α-emission rate of 100 µg of each sample for 24 hours was predicted by considering their isotopic composition and known specific activity of each isotope.

Sample ID	U-234	U-235	U-236	U-238	α-emissions/ 24 hours/ 100 µg
DU U_3_O_8_	0.000774(26)	0.2029(10)	0.00294(5)	99.793(1)	1.24E + 05
NatU UO_3_	0.005632(89)	0.7519(40)	0.000155(13)	99.242(3)	2.24E + 05
HEU U_3_O_8_	0.9688(44)	90.754(39)	0.3476(18)	7.929(50)	2.01E + 07

### Particulate sizing

**Fig 1:**
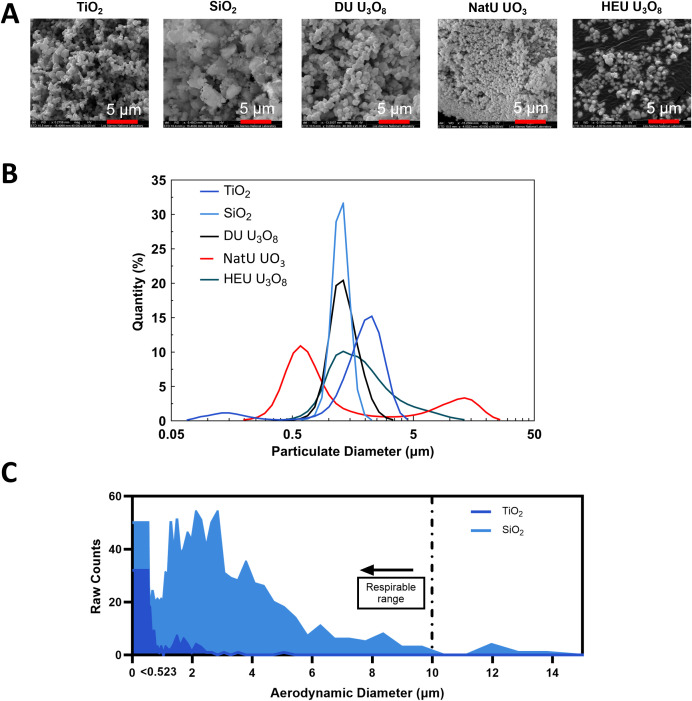
Characterization of particulates to calculate theoretical aerodynamic particulate size. **(A)** SEM images of all particulates for shape factor (*X*) estimation with a red scale bar of 5 µm. **(B)** DLS analysis of hydrodynamic diameter of all particulates shows majority within the human respirable range, < 10 µm MMAD. Dark blue = TiO_2_, light blue = SiO_2_, black = DU U_3_O_8_, red = NatU UO_3_, and green = HEU U_3_O_8_. **(C)** Experimental aerodynamic particulate size of TiO_2_ and SiO_2_ to compare theoretical results.


Radiological and control particulate scanning electron microscopy (SEM) images were collected on a FEI Quanta 200F using field emission with accelerating voltages of 10–30 kV from gold-coated samples (Fig 1A). The optical diameters were established by dynamic light scattering (DLS) acquired with a Laser Scattering Particle Size Distribution Analyzer, Horiba LA-950 (Fig 1B). The size and morphology (shape factor, *X*) of TiO_2_ and SiO_2_ particulates were analyzed using established protocols of SEM, DLS, aerodynamic particulate sizing (APS), and optical particulate sizing (OPS) shown in Fig 1C using Eq. (1). Due to APS and OPS instruments being in a non-radiological space, the aerodynamic diameter of the uranium oxide particulates was theoretically calculated by the experimentally determined aerodynamic diameters of the non-radiological particulates.

$$D_{AE}=D_{IO}\sqrt{\frac{\rho_{p}}{\rho_{\text{0}}X}}$$ (1)

D_AE_ is the theoretical aerodynamic particulate equivalent diameter, D_IO_ is the measured irregular particulate diameter found using DLS, ρ_B_ and ρ_0_ are the bulk particulate density measured from the mass-to-volume ratio of loose powder and reference unit density of an example water droplet falling at terminal velocity (ρ_0 _= 1 g/cm^3^), and *X* is the shape factor of the irregular particulate estimated by assessing the relative morphology of the particulates in the SEM images translating to literature values (Fig 1) [53]. All particulates showed a 3/2/1 cuboid shape factor (median = 1.745) except SiO_2_, which showed a 3/2/1 ellipsoid (median = 1.36). Details of equivalent aerodynamic particulate diameters can be found in the supplemental information (S3 Fig, S4 and S5 Tables in S1 File). All particulates used for these experiments were shown in Table 2 to be respirable to humans (<10 µm MMAD).

**Table 2:** Determination of aerodynamic equivalent particulate diameters through theoretical estimation. Radiological particulate aerodynamic equivalent diameters (D_AE_) were theoretically estimated using experimentally determined irregular aerodynamic diameters (D_IA_) of non-radiological particulates, TiO_2_ and SiO_2_, along with DLS irregular optical diameters (D_IO_), bulk densities (ρ_B_), and SEM-derived shape factors (*X*) from all respective particulates, and the reference density of water (ρ_0_) with Eq. (1).

	Aerodynamic diameters, D_IA_		Optical diameter sizes, D_IO_					
Quantity	TiO_2_	SiO_2_	TiO_2_	SiO_2_	DU U_3_O_8_	NatU UO_3_	HEU U_3_O_8_	
D_IO_ (µm)	1.54	2.08	1.828	1.173	1.224	0.802	1.583	Measured irregular particulate diameters
ρ_B_ (g/cm^3^)	0.77	2.32	0.77	2.32	8.3	8.3	8.3	Bulk powder density
ρ_0_ (g/cm^3^)	1	1	1	1	1	1	1	Reference density
χ, unitless	1.745	1.36	1.745	1.36	1.745	1.745	1.745	Shape factor from online source [53]
D_AE_ (µm)	1.02	2.72	1.21	1.53	2.67	1.75	3.45	Aerodynamic equivalent diameter

### Cell culture

Human adenocarcinoma lung epithelial cells, A549 (ATCC CL-185^TM^), were cultured in tissue-treated T75 vented cap flasks (Corning^®^, 3290) with Ham’s F-12 (Kaighn’s Modification) with L-glutamine and without phenol red media (Caisson, HFL12) supplemented with 10% (v/v) Fetal Bovine Serum (FBS, F2442) from Sigma Aldrich at 37°C, 5% CO_2_ in a humidified incubator (Benchmark Scientific, H3565-180). After 3 passages of reaching ~80% confluence, the A549s were seeded into a 96-well plate (Corning^®^, 3599) at 20,000 cells/well and incubated for 24 hours.

Primary human bronchial/tracheal epithelial cells (HBTEC, FC-0035, LOT #08265, LifeLine Cell Technologies^®^) and primary human small airway epithelial cells (HSAEC, FC-0016, LOT# 01434, LifeLine Cell Technologies^®^) were selected from healthy human donor lots. Each cell line was grown in T75 vented cap flasks (Corning^®^, 3290) with Lifeline^®^ BronchiaLife™ Epithelial Airway Medium with Life Factors till ~80% confluence by changing the media every other day. The cells were then expanded in T175 cm^2^ vented cap flasks (Corning^®^, CLS3292-50EA) again till ~80% confluence and cryopreserved. After 3–5 passages from the original manufactured vial, the cells were then seeded in a 96-well plate (Corning CellBIND^®^, 3300) at 40,000 cells/well and incubated for 3 days, changing the media every day.

### Cell viability and damage analysis

200 µL of varying concentrations of pre-sonicated (~20 minutes) uranium oxide and control particulates were added to each cell line. The particulate concentrations were 0.1, 1, 10, 50, 100, and 500 µg/mL. Cells were exposed to particulates in triplicate in each experiment, and each experiment was repeated four times for a total of 12 replicates. A positive cell damage control of 0.03% H_2_O_2_ was implemented, as well as an untreated cell negative control group.

The cell viability of each cell line was assessed by using water-soluble tetrazolium salt (WST-8) (Sigma Aldrich, 92992) and lactate dehydrogenase (LDH) activity assays (Sigma Aldrich, MAK066-1KT) using a plate reader (BioTek Synergy HTX). To assess a potential mechanism of cell death, a superoxide dismutase (SOD) assay (Sigma Aldrich, CS0009-1KT) was performed according to kit instructions, except for a 1:2 phosphate buffer saline (PBS) dilution of SOD product in each well before being read in the plate reader (BioTek Synergy HTX).

### Statistical analysis

Due to the smaller sample size (n = 4), the statistical differences in cellular response were determined using the Mann-Whitney non-parametric test. The adjusted p-value was determined with the Bonferroni-Dunn method. Statistical relevance was determined by having a p-value less than 0.05 and was further categorized by <0.01, < 0.001, and <0.0001 p-values, respectively. The degree of variance in each biological replicate (n = 4) was similar to the variance of total technical replicates (N = 12), allowing for the assumption to treat each of the 12 replicates for each particulate exposure as individual observations applied to the Mann-Whitney non-parametric test. For statistical comparison, the Mann-Whitney test results were compared to unmatched t-tests with a Welch correction using the Holm-Sidak method and showed no difference to the non-parametric test. Graphs were designed in GraphPad Prism v.10, and illustrations were designed in BioRender.

### Ethics statement

The Los Alamos National Laboratory Human Subjects Research Review Board conducted an administrative review of the project prior to work and did not require a full review or assign a study number because the work was not considered human research. The NIH Human Subjects Research Decision Tool result of this study was “not considered human subjects research” since all specimens and data were obtained from deceased individuals.

## Results

### Toxicity assessment (LC(50)) in lung cells exposed to uranium oxides

**Fig 2:**
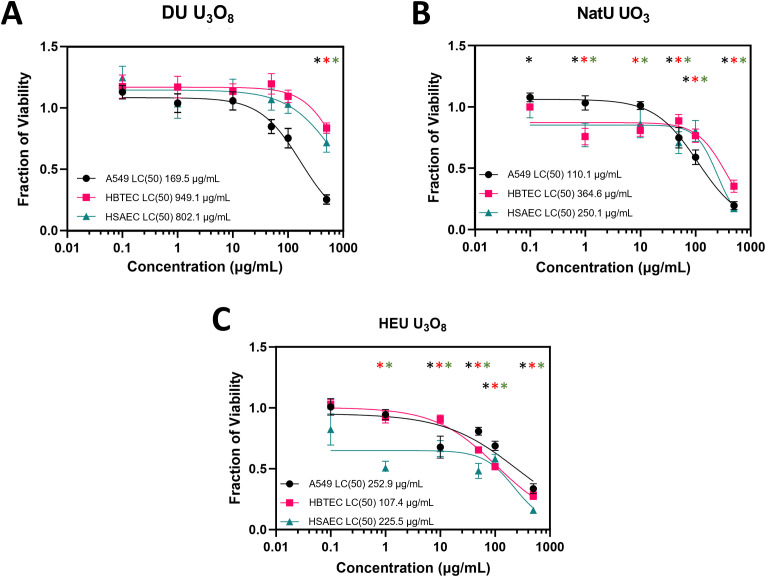
LC(50) curves of the effects of uranium particulates at differing enrichment values on A549, HBTEC, and HSAEC. WST-8 assay: **(A)** LC(50) of DU U_3_O_8_ on A549 (169.5 µg/mL), HBTEC (949.1 µg/mL), and HSAEC (802.1 µg/mL). **(B)** LC(50) of NatU UO_3_ on A549 (110.0 µg/mL), HBTEC (364.6 µg/mL), and HSAEC (250.1 µg/mL). **(C)** LC(50) of HEU U_3_O_8_ on A549 (252.9 µg/mL), HBTEC (107.4 µg/mL), and HSAEC (225.5 µg/mL). All values after 24-hour exposure. Black = A549, red = HBTEC, and green = HSAEC. Colored asterisks (*) above data points indicate a significant fraction of viability (p < 0.05) compared to normalized untreated cells (fraction of viability = 1.0) respective of each cell line; biological replicates, n = 4 and technical replicate total, N = 12 for each condition.

Each of the cell lines tested, A549, HBTEC, and HSAEC, displayed dose dependent toxicity when exposed to different uranium oxide isotopes. Lethal concentrations for 50% cell death (LC(50)) curves demonstrate the impact of increasing concentrations of uranium oxide on each type of cell, as compared to surrogate material (Fig 2 and S4 Fig in S1 File). The LC(50) value was determined by plotting the normalized absorbance against uranium oxide concentration and fitting a sigmoidal curve. Lower LC(50) values demonstrate a greater cytotoxic effect of the uranium oxides on the cells. A549s demonstrated the lowest LC(50) of 169.5 µg/mL following exposure to DU U_3_O_8_, while HBTEC and HSAEC showed higher values of 949.1 µg/mL and 802.1 µg/mL, respectfully. LC(50) associated with NatU UO_3_ indicates that A549 are most susceptible to changes in metabolism (110.0 µg/mL), followed by HSAEC (250.1 µg/mL) and HBTEC (364.6 µg/mL). We hypothesized that HEU U_3_O_8_ would have the smallest LC(50) amongst the different isotopes due to its increased α-radiation activity, thereby causing more cellular damage. This hypothesis was confirmed in the primary cell lines HBTEC (107.4 µg/mL) and HSAEC (225.5 µg/mL). A549s showed greater resilience to HEU U_3_O_8_ with an LC(50) value at 252.9 µg/mL. Thus, biological outcomes in immortalized cell lines vary from primary ones and may not be truly reflective of physiological systems.

#### Assessment of biological impact.

**Fig 3:**
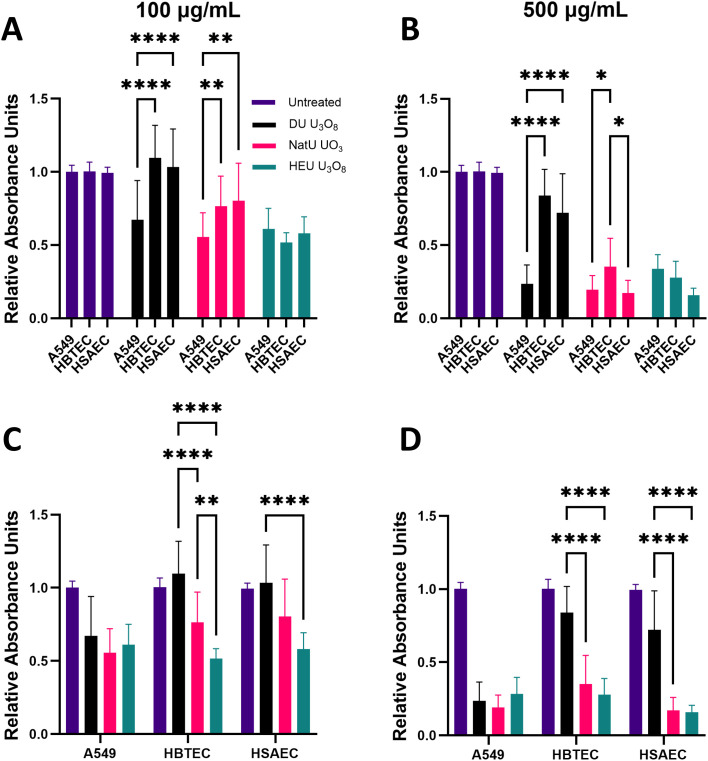
Assessing cell viability with WST-8 assay. Normalized A549, HBTEC, and HSAEC were exposed to 100 and 500 µg/mL of DU U_3_O_8_ (black), NatU UO_3_ (red), and HEU U_3_O_8_ (green) for 24 hours. Normalized viability differences between cell lines exposed to (A) 100 µg/mL and (B) 500 µg/mL of uranium oxide particulates; A549 shows significantly decreased viability compared to HBTEC (p = 0.0047) and HSAEC (p = 0.0026) at 100 µg/mL. HBTEC and HSAEC showed significantly decreased normalized viability with exposure to NatU UO_3_ (p < 0.0001) and HEU U_3_O_8_ (p < 0.0001) ((C) 100 µg/mL and (D) 500 µg/mL), compared to DU U_3_O_8_. An asterisk (*) indicates a significant fraction of viability: * (p < 0.05); ** (p < 0.01); *** (p < 0.001); **** (p < 0.0001) compared to normalized untreated cells (fraction of viability = 1.0); biological replicates, n = 4 and technical replicate total, N = 12 for each condition.

100 and 500 µg/mL of uranium oxide were selected for comprehensive bioanalysis because, based on the LC(50) values reported in Fig 2. All cell lines showed susceptibility to radioactive particulates at 500 µg/mL. At both 100 and 500 µg/mL, HBTEC and HSAEC demonstrated significantly increased cytotoxicity when compared to the untreated groups (Fig 3D), with both NatU UO_3_ and HEU U_3_O_8_. At 100 µg/mL, these two cell lines did not demonstrate susceptibility to DU U_3_O_8_ particulates. Of cells exposed to 100 µg/mL of NatU UO_3_ only HBTEC showed increased cytotoxicity (S5 Fig in S1 File). With exposure to 100 and 500 µg/mL of DU U_3_O_8_ particulates, A549 demonstrated the most significant decrease in viability (p < 0.001). No concentration dependence was measured in this cell line, and significant cytotoxic effects were observed in all exposure conditions compared to the untreated group (Fig 3C and 3D).

**Fig 4:**
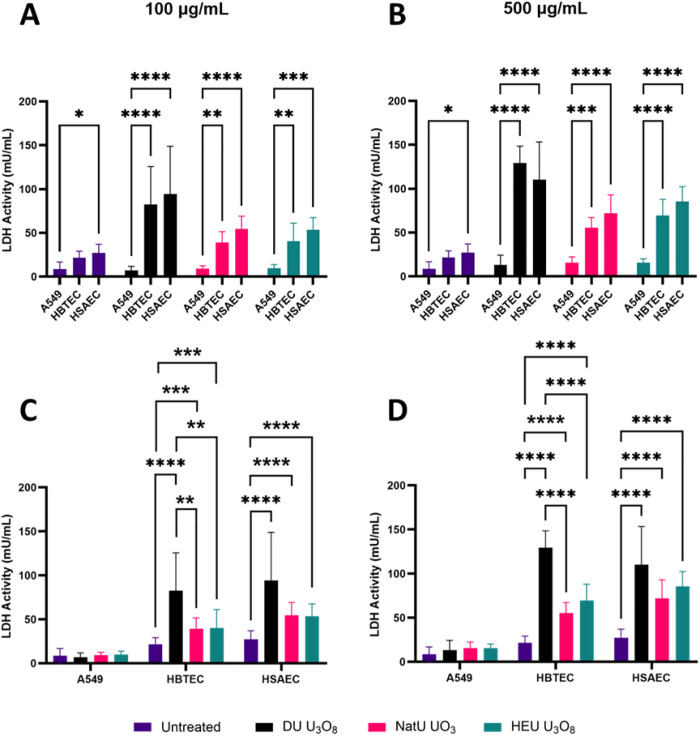
Measurement of LDH activity. LDH activity with 100 and 500 µg/mL of DU U_3_O_8_ (black), NatU UO_3_ (red), and HEU U_3_O_8_ (green) for 24 hours in A549, HBTEC, and HSAEC. Statistical differences were observed with (A) 100 µg/mL and (B) 500 µg/mL of uranium oxide particulates; A549 consistently showed lower LDH activity compared to HBTEC (p < 0.0001) and HSAEC (p < 0.0001). LDH activity was statistically different for each cell line after (C) 100 µg/mL and (D) 500 µg/mL of uranium exposure; A549 showed no relative changes in LDH activity, while both HBTEC and HSAEC showed increased LDH activity to all uranium oxide particulates. An asterisk (*) indicates a significant fraction of viability: * (p < 0.05); ** (p < 0.01); *** (p < 0.001); **** (p < 0.0001) compared to normalized untreated cells (fraction of viability = 1.0); biological replicates, n = 4 and technical replicate total, N = 12 for each condition.

Lactate dehydrogenase (LDH) activity was measured in the cell lines following exposure to uranium oxides as an indication of reduced cell membrane integrity in response to the emitted radiation and physiochemical properties of the particulates. LDH are intracellular enzymes that leak out of the cell once the membrane is compromised and can be detected to assess the severity of cellular damage [54]. A dose-dependent response was observed, indicating that higher concentrations of uranium oxide particulates compromised cell membrane integrity, a key indicator of apoptosis and viability (S7 Fig in S1 File). At 100 and 500 µg/mL of all particulates, LDH activity in both HBTEC and HSAEC was significantly higher (p < 0.01) than in A549s (Fig 4A and 4B). HBTEC and HSAEC did not demonstrate significant differences between themselves. In the A549s, LDH activity was not significantly enhanced with either concentration of uranium (Fig 4C and 4D). LDH activity was significantly higher in primary cells exposed to DU U_3_O_8_ than in either NatU UO_3_ or HEU U_3_O_8_. The one exception to this was HSAECs exposed to HEU U_3_O_8_ (Fig 4C and 4D).

Superoxide dismutases (SODs) are protective antioxidant enzymes that are essential in the defense against reactive oxygen species (ROS) produced by ionizing radiation or cellular processes [55]. We measured SOD activity in the cell lines following exposure to uranium oxides as an indication of a protective response to radiation exposure.

**Fig 5:**
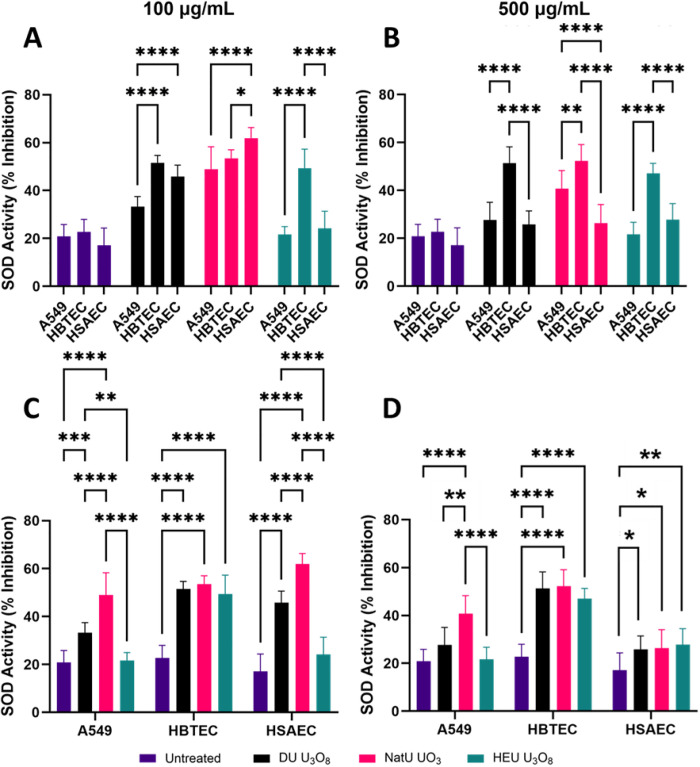
Measurement of SOD activity. SOD activity in A549, HBTEC, and HSAEC exposed to 100 and 500 µg/mL of DU U_3_O_8_ (black), NatU UO_3_ (red), and HEU U_3_O_8_ (green) for 24 hours. (A) 100 µg/mL and (B) 500 µg/mL exposures to uranium oxides resulted in statistically significant induction of SOD activity. SOD activity with uranium oxide particulates for each cell line at (C) 100 µg/mL and (D) 500 µg/mL. An asterisk (*) indicates a significant change in SOD release: * (p < 0.05); ** (p < 0.01); *** (p < 0.001); **** (p < 0.0001) compared to normalized untreated cells (fraction of viability = 1.0); biological replicates, n = 4 and technical replicate total, N = 12 for each condition.

HBTECs showed the highest SOD activity when exposed to any of the particulates and at either concentration, while A549s had the lowest (Fig 5). All cell lines exhibited increased SOD activity when exposed to DU U_3_O_8_ and NatU UO_3_, except A549s when exposed to 500 µg/mL of DU U_3_O_8_. HEU U_3_O_8_ did not induce a significant response in A549 and HSAEC, except when the latter was exposed at 500 µg/mL concentration (Fig 5C and 5D).

## Discussion

Biological systems are extremely complex, and cell systems do not often effectively replicate this physiology. *In vitro* cultured cells respond differently to particulates based on type of cell, type of particulate, other factors such as oxidation state, and concentrations. Understanding the factors that contribute to the variability in cellular response to particulates can allow us to develop or integrate physiologically relevant *in vitro* models, which is our goal. In this manuscript, we begin to address this challenge via the simultaneous and systematic comparison of the response to distinctive uranium particulate enrichments (DU, NatU, and HEU) across varied lung cell (A549, HBTEC, and HSAEC). We assessed cell viability, health, and response over 24 hours via measurement of WST-8, LDH, and SOD activities in these cells. While our results reaffirmed that there is a significant difference in the response of different cells to uranium oxide particulates based on the type of enrichment, concentration, and oxide state, it also shed some light on the mechanisms and processes involved in these differences. Our outcomes strongly indicate the differences in the response between primary and immortalized cell lines, suggesting that the choice of the cell line should be carefully made, and mechanistic insights are required to evaluate the relevance of these differences in the physiological context. Thus, the continued systematic study of cell and tissue behavior in the context of radiological exposure is needed in order to provide data for toxicological analyses benchmarking their responses to *in vivo* human or animal data. Modern improvements of data availability and artificial intelligence (AI) models allow complex insights and detailed comparisons to translate results from *in vitro* to more physiologically relevant *in vivo* exposures, for instance accidental human exposures and animal studies [56]. AI models could tackle the fundamental challenge to extrapolate simplified *in vitro* models to more complicated *in vivo* models by accounting for the missing biological components presented in a whole organism and better understand dose responses. Our hope is that the *in vitro* results provided would be an initial step for these advanced AI comparisons to improve radiation protection strategies and risk assessments.

General viability of all cell lines was inhibited at some capacity in response to each of the uranium oxide particulates tested, and this response was concentration dependent as well. The U_3_O_8_ particulates had low (DU) and high (HEU) isotopic ratio samples to understand the effects of increasing α-radiation activity on cells. Exposure to HEU U_3_O_8_ particulates significantly decreased HBTEC and HSAEC viability and proliferation compared to DU U_3_O_8_, as measured by the WST-8 cells (Fig 3). Conversely, A549s were sensitive to both DU and HEU U_3_O_8_ particulates, but the increased isotopic ratios did not magnify toxicity, as the measurements were not statistically different under these two exposure conditions. Since A549s demonstrated equal toxicity to all tested isotopic contents, it is probable that they are more sensitive to the uranium particulates—that is, *via* chemical properties than radiological toxicity—but further testing is required to justify this claim. The reduced response of primary cell lines to DU could potentially be due to their more stabilized genome, which may contribute to protection against radiological insult and/or heavy metal toxicity [57]. In addition, the differences between immortalized and primary cells in cell death and repair gene expression may account for some of the differences seen in the current study [58]. The number of cellular passages can also affect phenotypic results and genes related to the cell cycle that are expressed [59].

The fissile U-235 content of natural UO_3_ (0.7519%) is not dramatically different from that of depleted U_3_O_8_ (0.2029%). Thus, α-radiation activity over 24 hours is relatively similar in both samples (Table 1). UO_3_ has a greater number of moles per gram of uranium than U_3_O_8._ This is accounted for in the emissions calculations for Table 1. However, viability and proliferation when exposed to NatU UO_3_ were consistently lower as compared to the DU U_3_O_8_, a result especially seen in the primary cell lines. This may be associated with the comparatively higher presence of U-234 with its high-energy daughters, enhanced solubility, and consequent bioavailability of the isotope. UO_3_ and U_3_O_8_ are mostly insoluble in water, but UO_3_ solubility has been observed to be greatly enhanced in solutions with inorganic salts, such as NaCl, KCl, and KNO_3,_ suggesting higher solubility in cell media [32,60]. The soluble form of UO_3_, uranyl hydroxide (UO_2_(OH)_2_), can potentially cause more robust internal damage due to its ionic complexation with cell membrane permeable proteins [61]. Thus, the oxidation state of uranium particulates could modulate toxicological responses based on their solubility even at 24 hours of exposure. A systematic study of toxicology as a function of particulate solubility can help derive more robust inferences.

In assessing cellular respiration via LDH activity, no increase was evidenced in A549s for any of the uranium oxides, even though significant enhancement was noted in the primary cell models. The physiological makeup of immortalized versus primary cell lines results in disparate outcomes in such studies and must be better characterized to ensure relevance. LDH activities following exposure to depleted U_3_O_8_ were significantly higher for both primary cell lines compared to those associated with NatU UO_3_ and HEU U_3_O_8_. Whereas this could be truly indicative of the biological interactions being assessed, it is important to rule out artifactual factors such as the influence of media composition, among others. For instance, the media used for primary cell cultures is serum-free and produced a small amount of precipitate when the uranium particulates were added, whereas the A549 serum-containing media showed no sign of complexation. Controls, such as PBS with uranium oxides and both serum and serum-free cell media with uranium oxides without cells, were implemented to account for the possibility that the formed precipitate produced scattering of the plate reader laser and skewed the absorbance values. We subtracted values from wells that contained only uranium oxides and serum-free media, but it is uncertain whether the additional cell interaction with the oxides magnified scattering of the beam. A possible explanation for the formation of the precipitate is that the ionic form of the uranium oxides, uranyl (UO_2_^2+^), complexed with the anionic species present in the serum-free primary media. Specific fats and proteins from the A549 serum-containing media could have interrupted any uranium precipitates from forming. Further investigation into this speciation of uranium in serum- and non-serum-containing media—as well as common cell culture additives like antibiotics—is currently ongoing in our laboratory by using ultraviolet and Raman spectroscopy to identify the components that contribute to precipitation. By comparing the Raman spectra of serum and serum-free media with uranium oxide, a molecular fingerprint can be identified. Another possibility is that the LDH release was inactivated by certain unknown reactive chemicals in the A549 media or mixture of media with the uranium particulates, causing low levels of LDH [62]. The addition of 10% FBS to the A549 media is known to show increased LDH levels, which is contradictory to what was seen in the results [63]. The cellular mechanisms as to why DU U_3_O_8_ significantly increased LDH activity compared to HEU U_3_O_8_ in primary cells are notable and will be investigated in future projects.

SOD activity generally increased across all cell models and uranium oxides compared to untreated controls. Upon exposure to HEU U_3_O_8_, HBTECs showed significantly elevated SOD activity compared to either A549 or HSAEC models. This is one of the few cases wherein a difference in activity was observed among the primary cell types. HBTECs were the only cell model that expressed significantly elevated SOD activity to each uranium oxide particulate at 100 and 500 µg/mL. At 100 µg/ml, no SOD activity was evidenced in HEU U_3_O_8_, which did not elicit an increase in SOD activity in A549 or HSAEC, indicating that it takes a more significant exposure risk to induce protective mechanisms in these *in vitro* models. The mechanism as to why DU U_3_O_8_ was able to cause increased SOD activity compared to HEU U_3_O_8_ in the smaller airway cell models may relate to higher SOD response as a protective mechanism in the upper airway [64].

## Conclusions

Our studies begin to shed light on mechanistic insights into the properties of cell-based systems and their suitability for studying exposure to uranium particulates. Each cell line responded differently to uranium particulates, further influenced by enrichment or oxidation state. Variations in oxidation states of the uranium particulates influenced viability across all cell lines, whereas increased enrichment corresponded to increased toxicity in the primary cells, HBTEC and HSAEC, but not for the immortalized A549. Primary upper respiratory tract cells, HBTEC, displayed decreased viability and increased SOD activity levels, correlating with the greatest general susceptibility to all radioactive particulates (DU U_3_O_8_, NatU UO_3_, and HEU U_3_O_8_). These findings indicate that a comprehensive assessment of cell system viability and the influence of underlying factors (media composition, stage of growth, confluence, type of particulate) is required before deeper conclusions on the impact to complex biosystems can be drawn from cell studies. However, with modern improvements of data availability and artificial intelligence models, these *in vitro* results can potentially be translated/compared to more physiologically relevant *in* vivo human exposures, alongside animal studies, to advance radiation protection strategies risk assessments, and countermeasure development. Future directions include the further disassociation of α-radiation from heavy-metal particulate toxicity to understand which mechanism is the underlying, or greater, cause of cellular death. Finally, systematic time-course evaluations to investigate prolonged dose response and investigations of the solubilities of different uranium oxides in cell media and the complexes they produce will be needed to create additional data sets that can be applied to translational toxicology studies in understanding how these cell types relate to human response.

## Supporting information

S1 File Characterization of particulates and additional controls. (DOCX)

S1 Fig Graphical abstract—Overview of the study design. Three different enrichment states and two oxide states of uranium particulates were separately introduced to three distinct submerged *in vitro* human epithelial lung cell lines to investigate if increased alpha emissions, oxidation state, or respiratory tract location causes differences in toxicological effect.
(TIF)
