# Supplementary material for: In vitro toxicity assessment of uranium particulates on different human lung epithelial cell models
Source: PLoS One. 2025 Oct 31;20(10):e0334247. doi: 10.1371/journal.pone.0334247 (PMC12578232; doi:10.1371/journal.pone.0334247)
Supplement: S1 File — (DOCX) [file pone.0334247.s001.docx]

*In Vitro* Toxicity Assessment of Uranium Particulates on Different Human Lung Epithelial Cell Models

**Supporting information: Characterization of Particulates and Additional Controls**

**
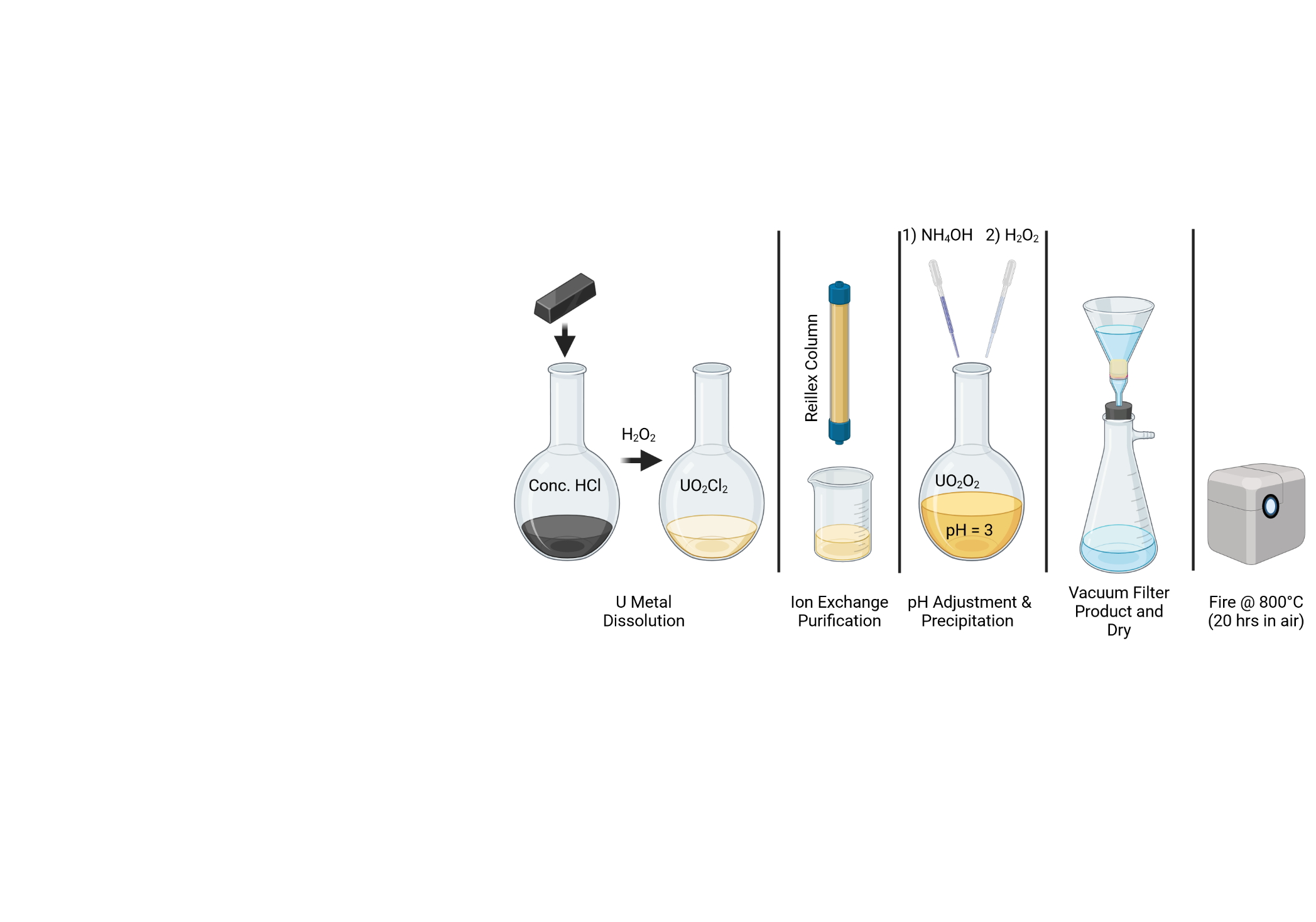
**

**S1 Fig. Elementary uranium oxide (U_3_O_8_) particulate synthesis diagram.** The synthesis of UO_3_ varied slightly by an increased firing time of 40 hours.


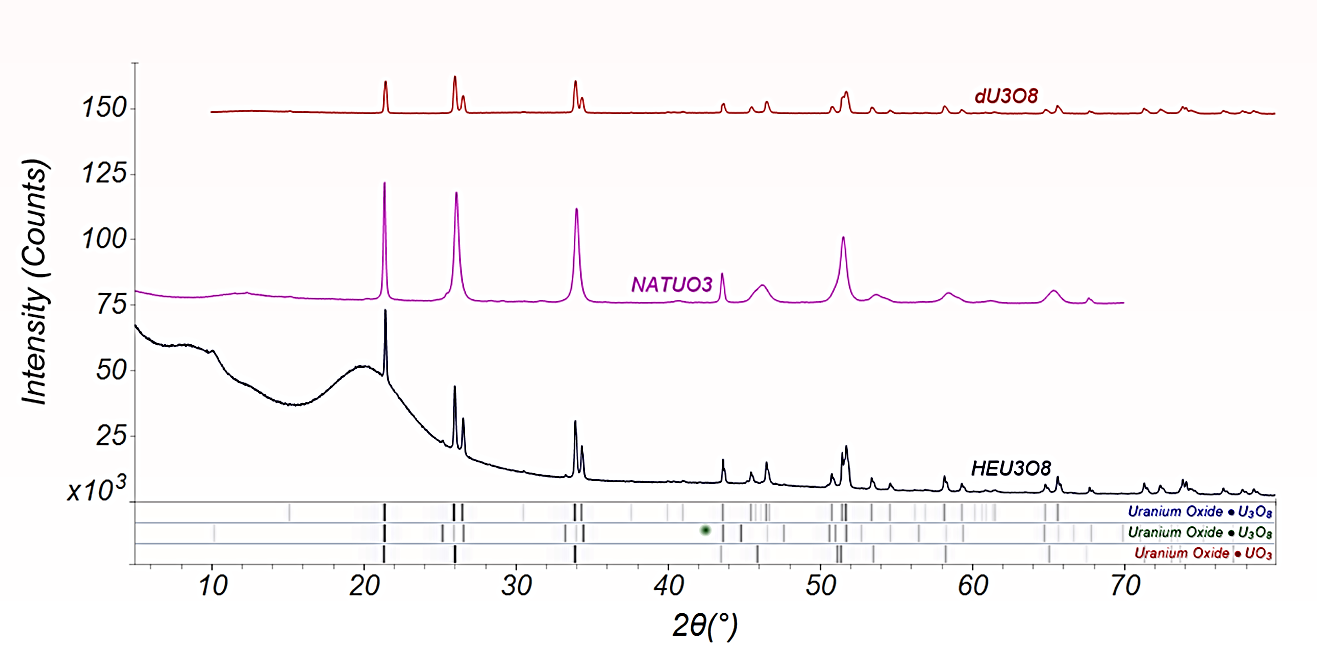


**S2 Fig. P-XRD analysis.** Red, depleted U_3_O_8_; Magenta, natural UO_3_; Black, highly enriched U_3_O_8_.

**Aerodynamic equivalent diameter from an aerodynamic sizer or an optical sizer**

For this study, it is helpful to define four particulates: an aerodynamically measured irregular particulate (S3a Fig), an optically measured irregular particulate (S3a Fig), a spherical water droplet of unit density (S3b Fig), and a Stokes equivalent sphere (S3c Fig).

**
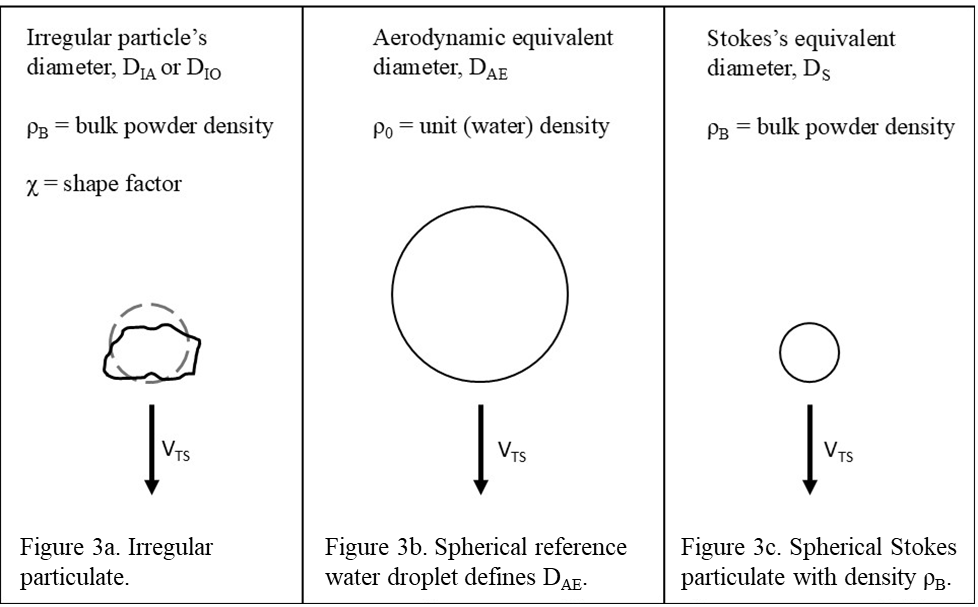
**

**S3 Fig. Difference in aerodynamic diameters.** Using Stokes’ guidelines for aerodynamic relationships, irregular and spherical particulates are compared and assigned variables to solve the theoretical aerodynamic particulate diameter of the radiological particulates.

If all four particulates exhibit the same magnitude of settling velocity (V_TS_) in still air, they are defined to have the same aerodynamic equivalent diameter (A_ED_),

$$V_{TS}= \frac{\rho_{B} D_{IA}^{2} g}{18 \mu\chi} = \frac{\rho_{B} D_{IO}^{2} g}{18 \mu\chi} = \frac{\rho_{0} D_{AE}^{2} g}{18 \mu} = \frac{\rho_{B} D_{S}^{2} g}{18 \mu}$$

S. Eq. (1)

where,

V_TS_ = terminal settling velocity of all three example particulates is equivalent,

ρ_B_ = bulk powder density measured from the mass to the volume ratio of loose powder,

ρ_0_ = unit density of an example water droplet falling at terminal velocity,

µ = air viscosity at ambient conditions,

χ = shape factor of the irregular particulate,

D_IA_ = “diameter irregular aerodynamic” (physical size of the particulate in an aerodynamic sizer),

D_IO_ = “diameter irregular optical” (physical size of the particulate in an optical sizer),

D_AE_ = “diameter aerodynamic equivalent” of the measured particulate,

D_S_ = “diameter Stokes”, and

g = gravitational acceleration.

If an irregular-shaped particulate is analyzed (i.e. in a TSI Inc model 3321 APS Aerodynamic Particulate Sizer) then the instrument output yields the aerodynamic equivalent diameter, D_AE_, when the ratio (ρ_B_/χ) is the input parameter for particulate density in the instrument software. If this is performed, then the TSI model 3321 APS does not need the D_IA_ or the ρ_0_ values to be specified. (The D_IA_ “diameter irregular aerodynamic” value is internally accounted for when the time-of-flight of the aerosol particulate is measured by a paired set of lasers).

However, if the irregular particulate is initially analyzed in the aerodynamic sizer with an input (i.e. the instrument’s default setting) value of ρ_0_ = 1.0 g/cc for the particulate density, then the aerodynamic equivalent diameter may be determined in a secondary step with equation (2) by using the initially determined D_IA_ value as the equation (2) input.

$$D_{AE}=D_{IA} \sqrt{\frac{\rho_{B}}{\rho_{0}\chi}}$$

S. Eq. (2)

However, when the diameter D_IO_ of an irregular-shaped particulate is optically measured (i.e. in a Horiba Laser Scattering Particulate Size Distribution Analyzer Partica LA-950V2) the D_IO_ “diameter irregular optical” value is used as an input to yield the D_AE_ value,

$$D_{AE}=D_{IO} \sqrt{\frac{\rho_{B}}{\rho_{0}\chi}}$$

S. Eq. (3)

These tabulated results assume the relationship between the aerodynamic and optical measurements are valid for estimating the aerodynamic equivalent diameters. If the particulates were spherical particulates with well-defined densities, these relationships would coincide.


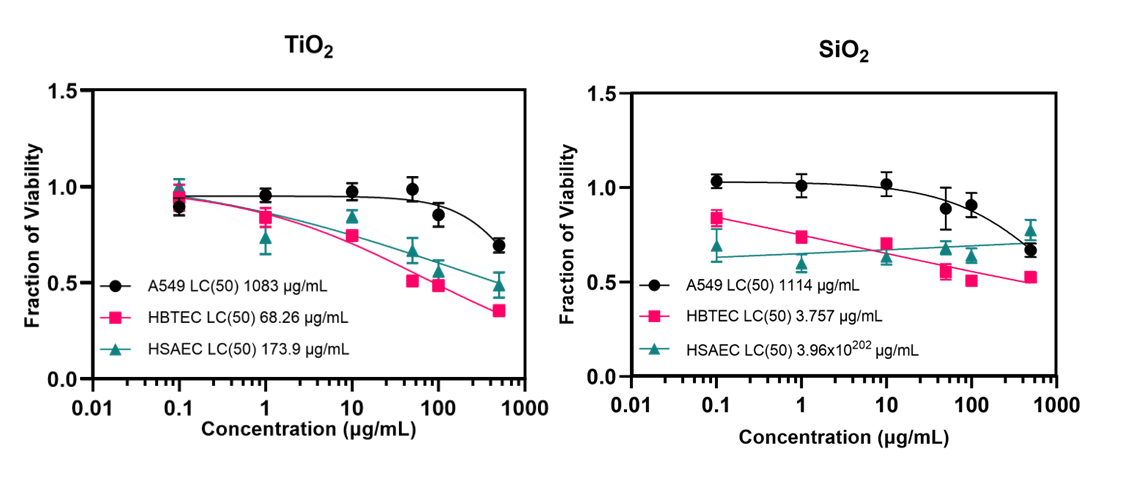


**S4 Fig. LC(50) curves for TiO_2_ and SiO_2_.** TiO_2_ on A549 (1083 µg/mL), HBTEC (68.26 µg/mL), and HSAEC (173.9 µg/mL); SiO_2_ on A549 (1114 µg/mL), HBTEC (3.757 µg/mL), and HSAEC (3.96x10E+202 µg/mL); biological replicates, n=4 and technical replicate total, N=12.


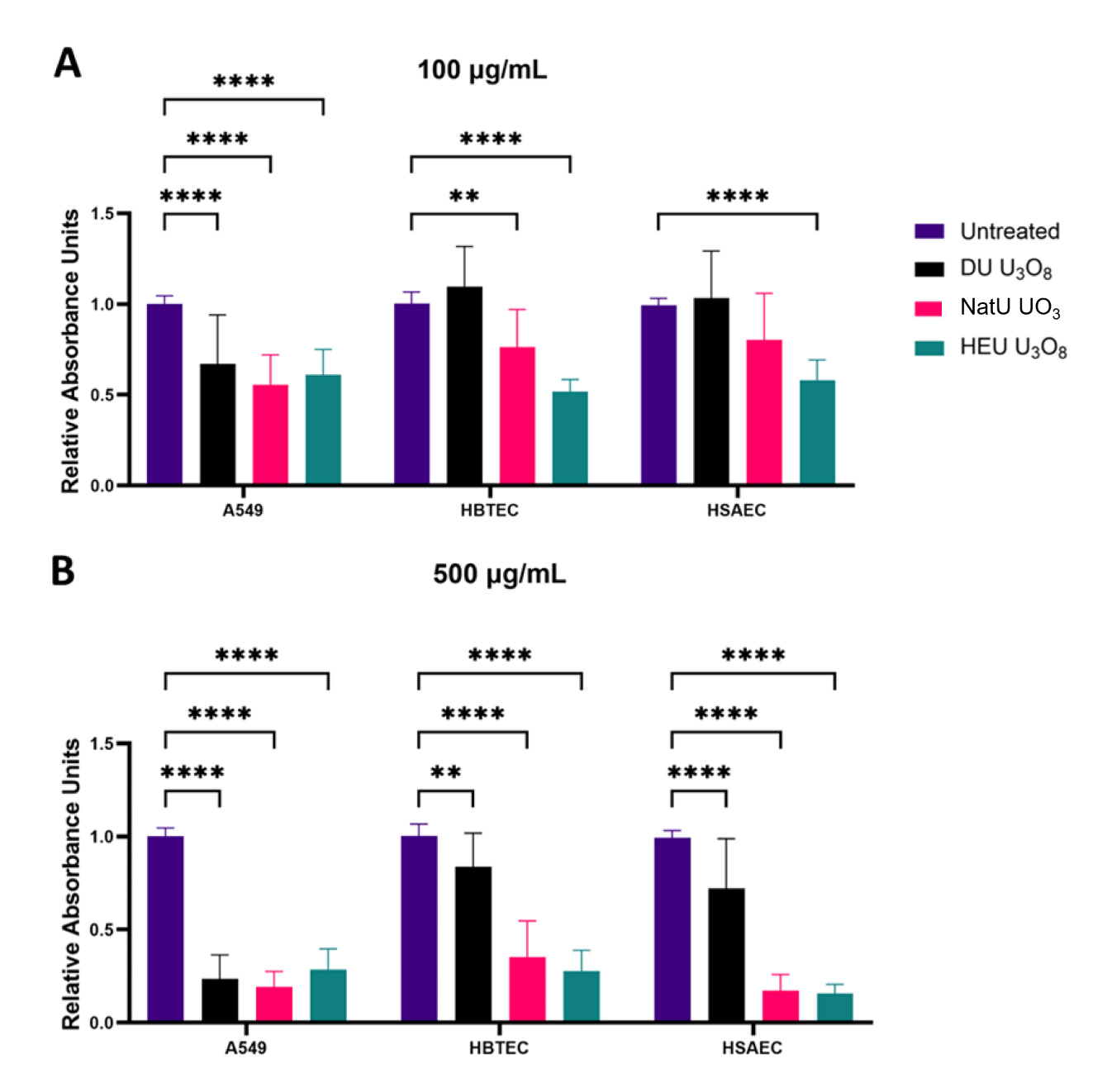


**S5 Fig.** **Viability analysis compared to untreated controls across cell lines exposed to 100 and 500 µg/mL of uranium oxide — WST-8 assay.** Viability compared to untreated control at (A) 100 µg/mL and (B) 500 µg/mL. Asterisk (*) indicates a significant fraction of viability, * (p <0.05); ** (p <0.01); *** (p <0.001); **** (p <0.0001) compared to normalized untreated cells (Fraction of viability = 1.0); biological replicates, n=4 and technical replicate total, N=12.

**
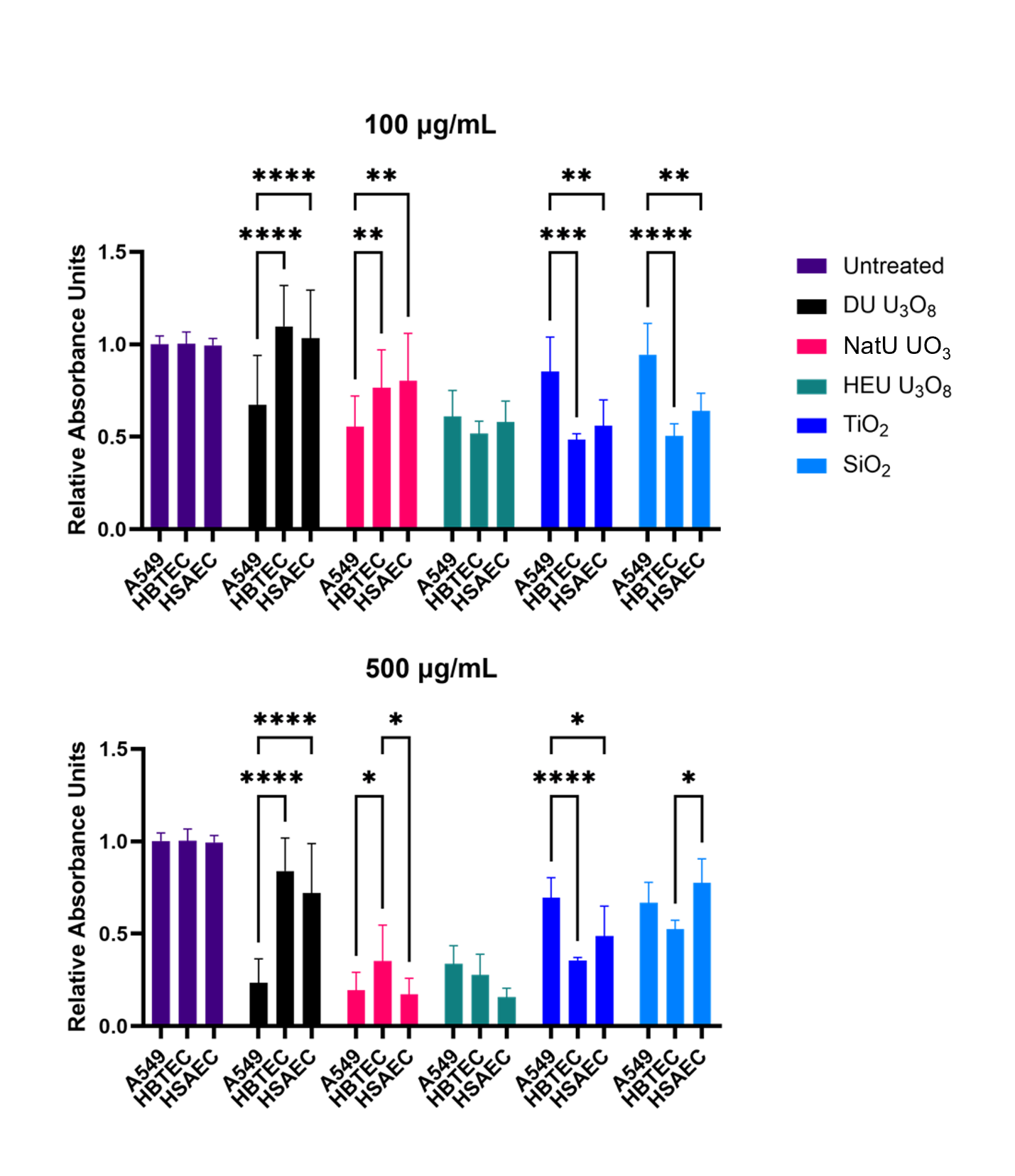
S6 Fig. WST-8 viability analysis across cell lines with 100 and 500 µg/mL of particulates.** Asterisk (*) indicates a significant fraction of viability, * (p <0.05); ** (p <0.01); *** (p <0.001); **** (p <0.0001) compared to normalized untreated cells (Fraction of viability = 1.0); biological replicates, n=4 and technical replicate total, N=12.


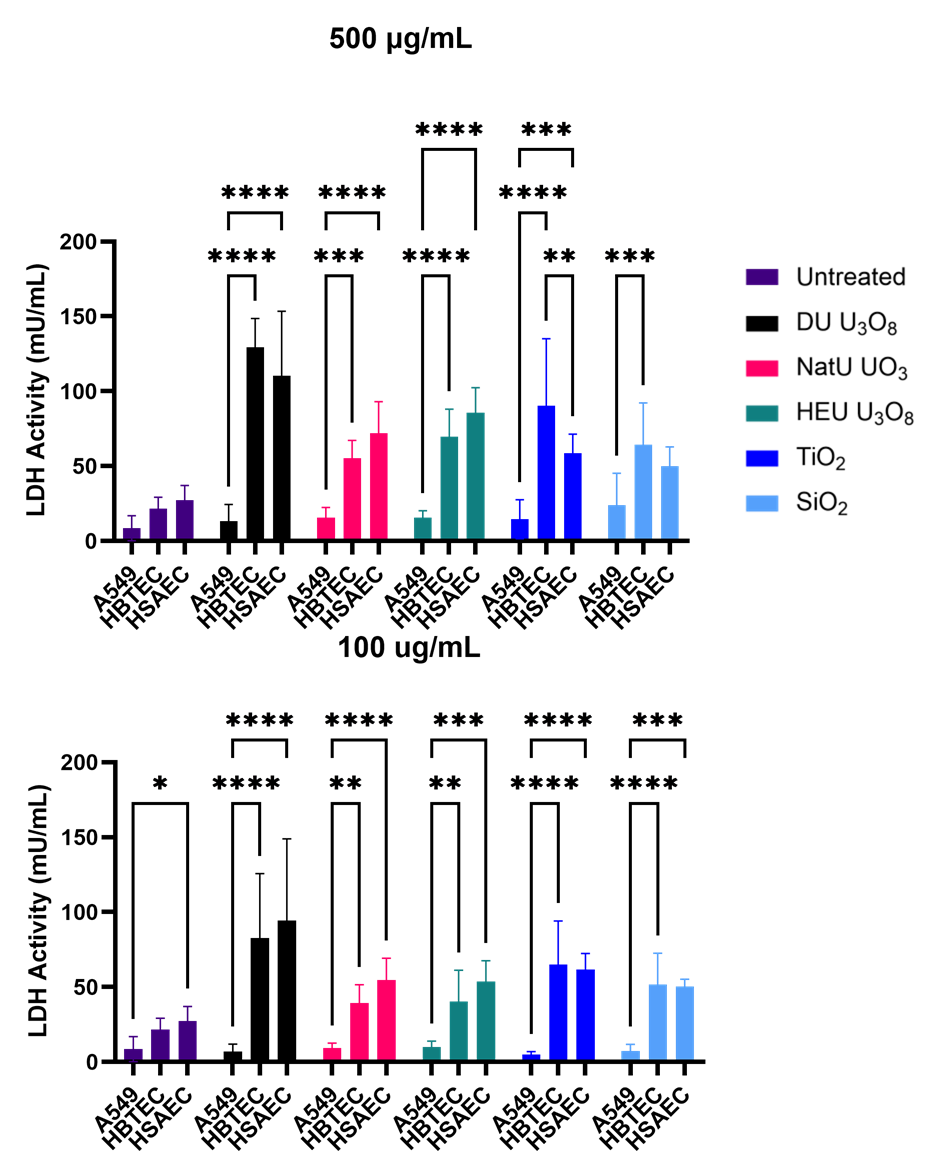

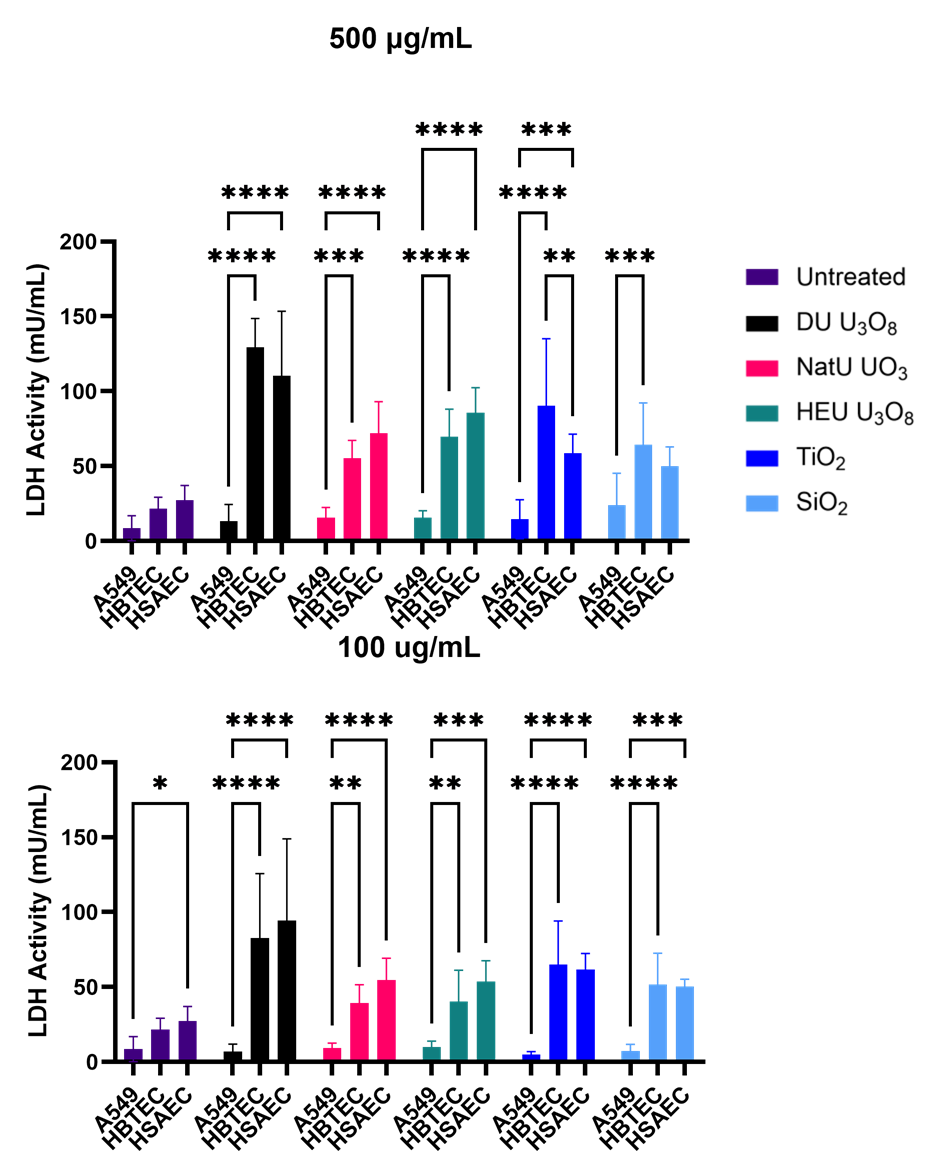

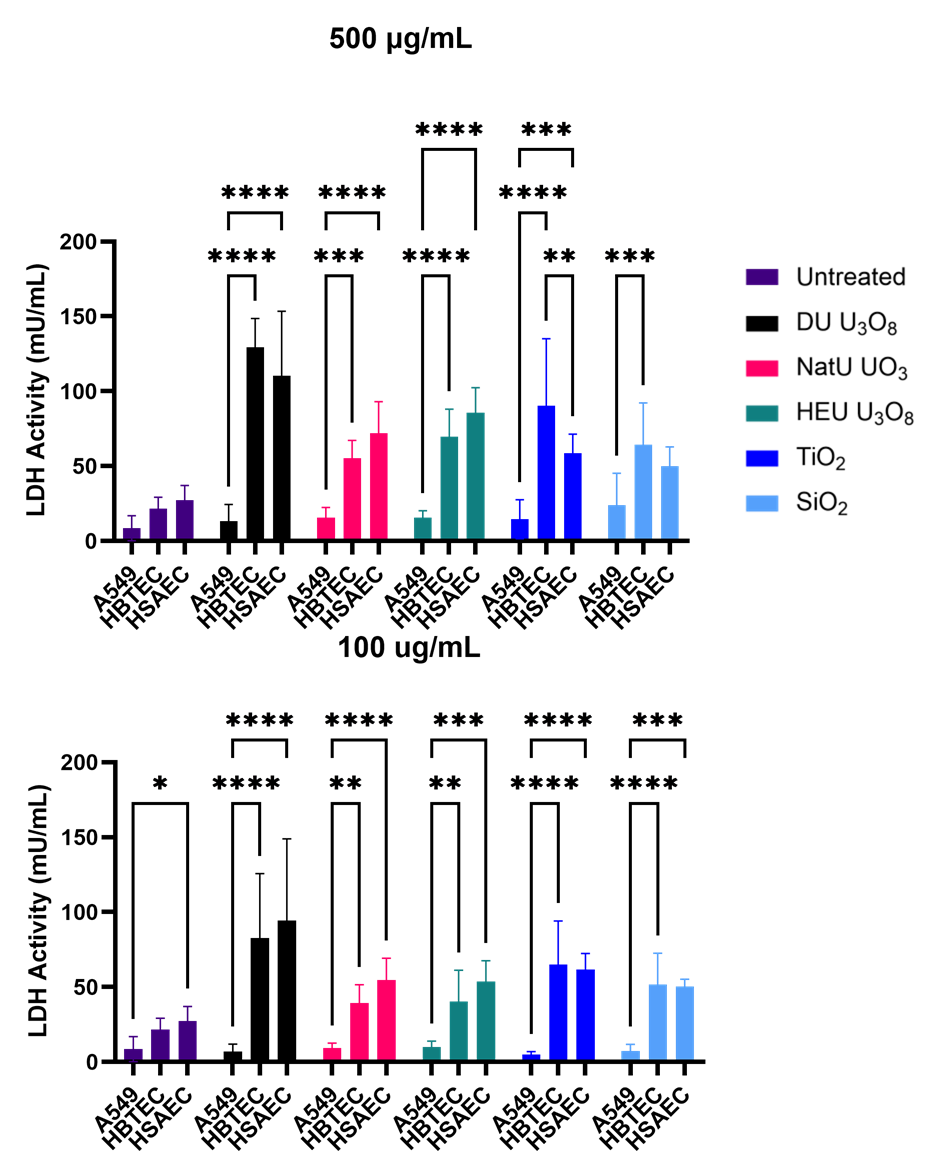


**S7 Fig. LDH activity compared across cell lines at 100 and 500 µg/mL of particulates.** Asterisk (*) indicates a significant fraction of viability, * (p <0.05); ** (p <0.01); *** (p <0.001); **** (p <0.0001) compared to normalized untreated cells (Fraction of viability = 1.0); biological replicates, n=4 and technical replicate total, N=12.

**
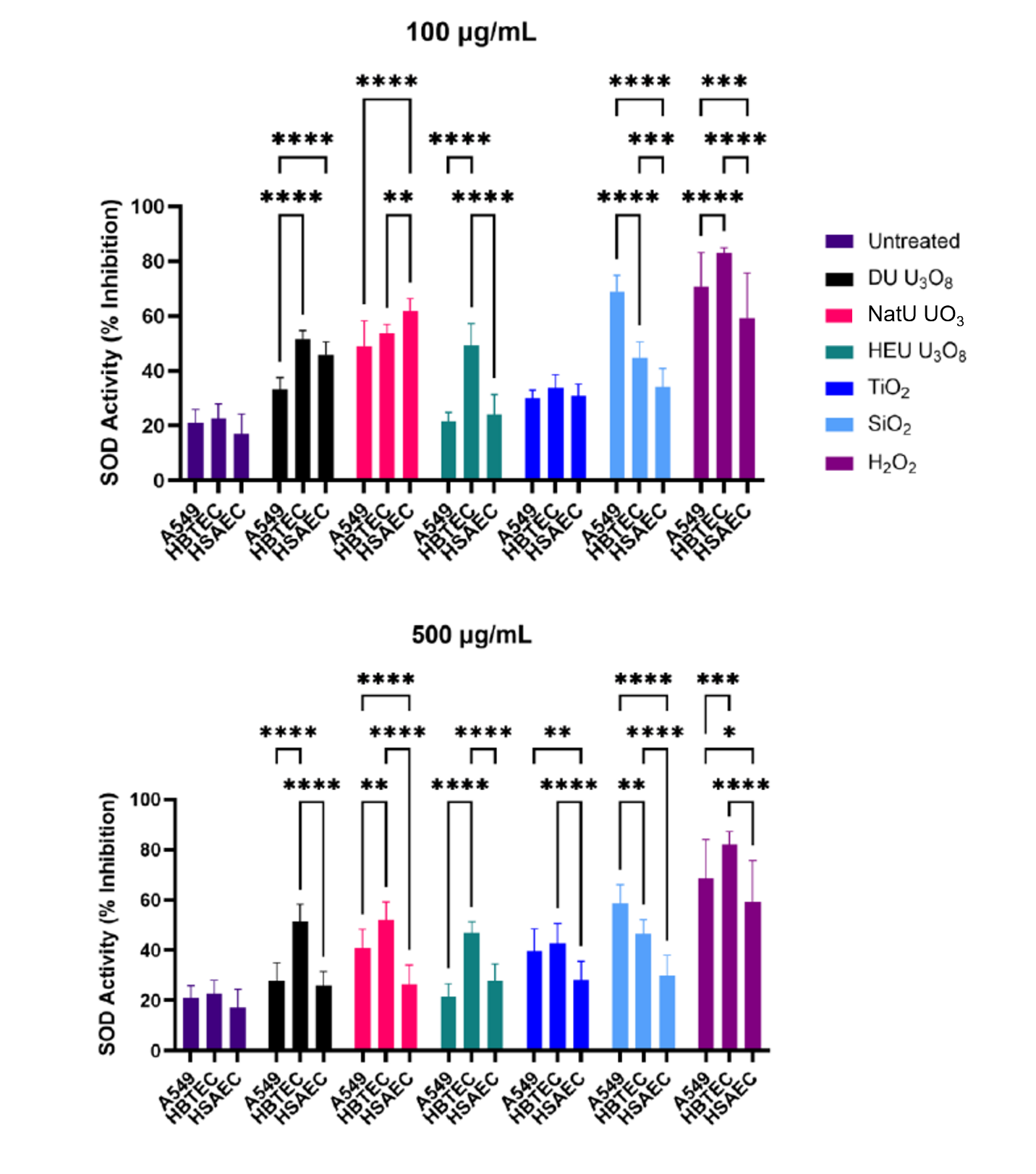
**

**S8 Fig. SOD activity compared across cell lines at 100 and 500 µg/mL of particulates and a 0.03% H_2_O_2_ positive control.** Asterisk (*) indicates a significant fraction of viability, * (p <0.05); ** (p <0.01); *** (p <0.001); **** (p <0.0001) compared to normalized untreated cells (Fraction of viability = 1.0); biological replicates, n=4 and technical replicate total, N=12.

**S1 Table. Atom ratios relative to ^235^U.** Atomic ratios of depleted uranium, U_3_O_8_ (GW DU), natural uranium, UO_3_ (GW NAT), and highly enriched uranium, U_3_O_8_ (GW HEU) relative to uranium-235 were determined using a quadrupole inductively-coupled plasma mass spectrometer with measurement uncertainty in parentheses. GW denotes the researcher initials. These are the same samples defined in the rest of the document simply as DU, NatU, and HEU.


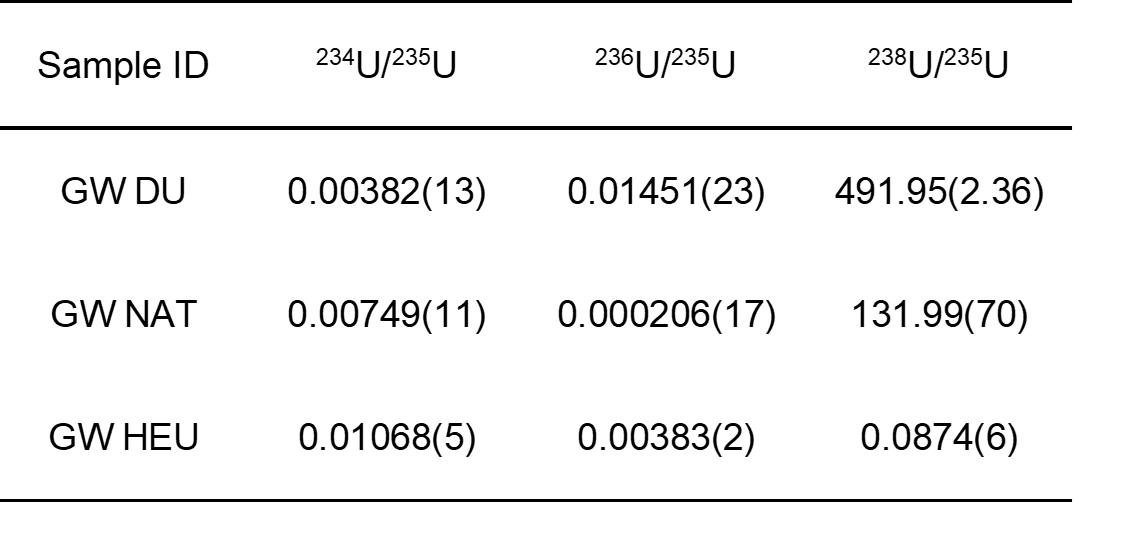


**S2 Table. Quality control: atom percent composition.** Standardized reference (ref) uranium material and our samples (obs) were compared using quadrupole inductively-coupled plasma mass spectrometer with measurement uncertainty in parentheses.


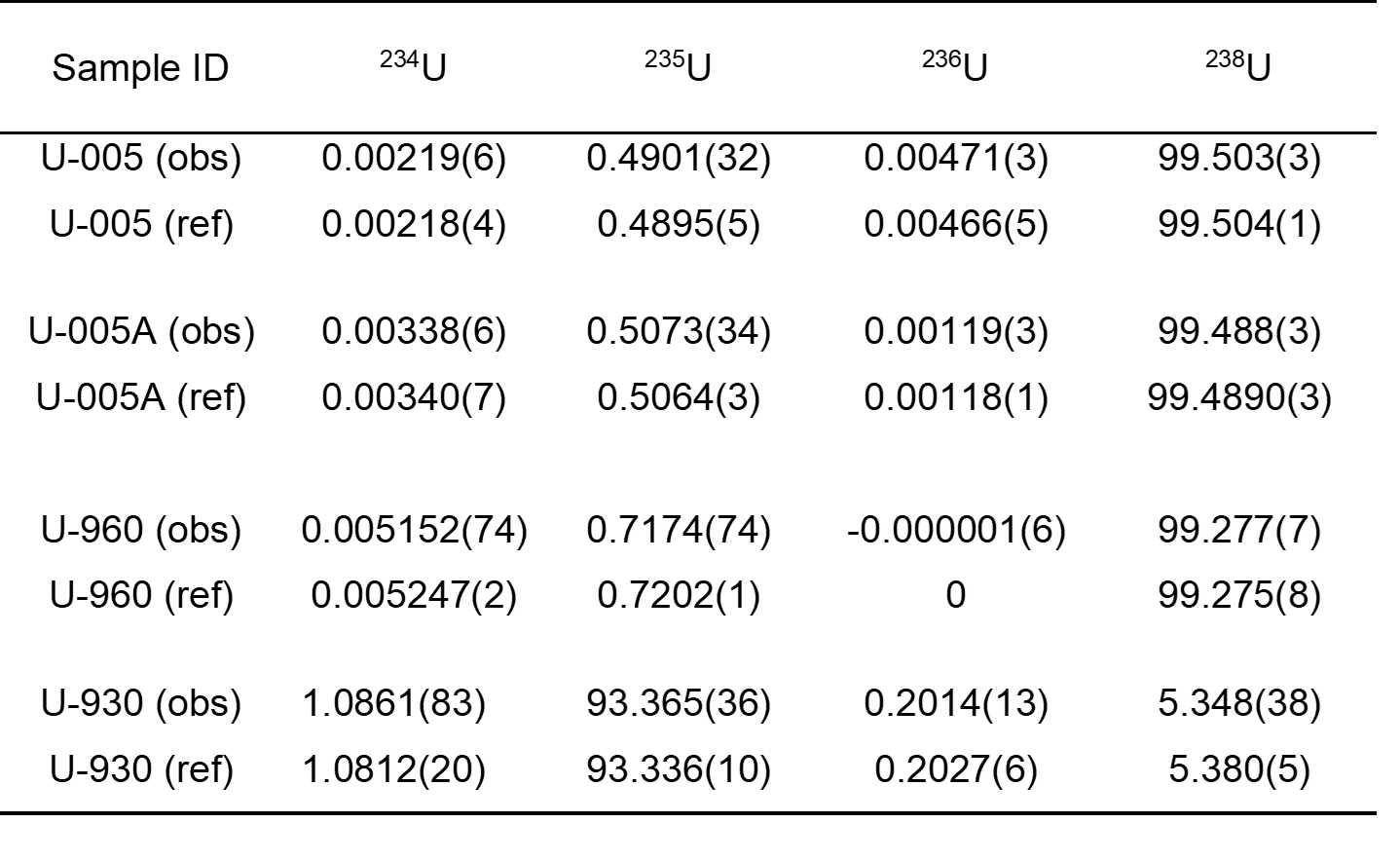


**S3 Table. Quality control: atom rations relative to ^235^U.** Standardized reference (ref) uranium material and our samples (obs) were compared using quadrupole inductively-coupled plasma mass spectrometer with measurement uncertainty in parentheses.


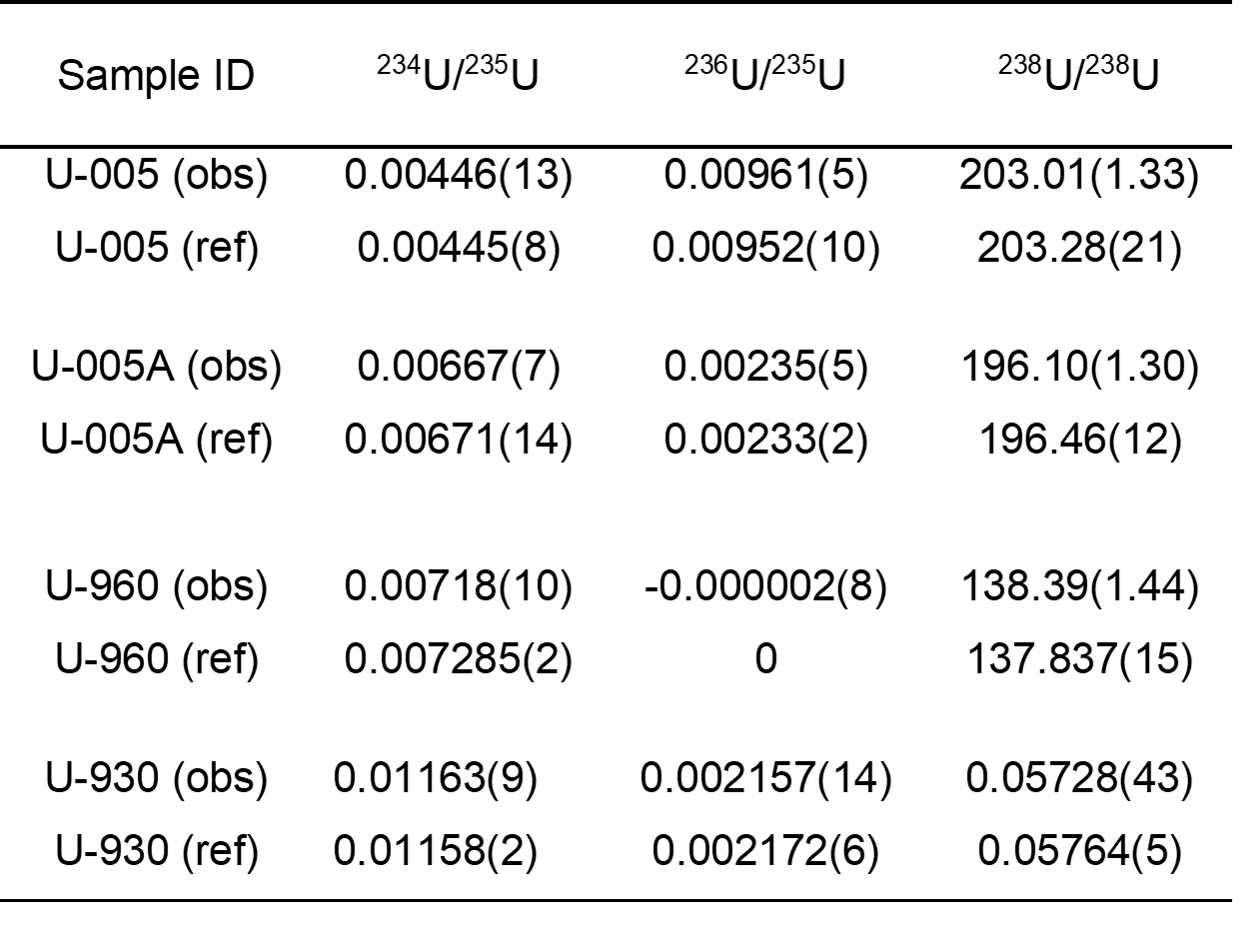


**S4 Table. Determination of aerodynamic equivalent particulate diameters.** Observed aerodynamic diameters were used to calculate aerodynamic equivalent particulate diameters using Eq.1 to ensure particulates were respirable by humans (< 10 µm). The units are: ρ_B_ = bulk powder density measured from the mass to the volume ratio of loose powder, ρ_0_ = unit density of an example water droplet falling at terminal velocity, χ = shape factor of the irregular particulate, D_IA_ = “diameter irregular aerodynamic” (physical size of the particulate in an aerodynamic sizer), D_IO_ = “diameter irregular optical” (physical size of the particulate in an optical sizer), and D_AE_ = “diameter aerodynamic equivalent” of the measured particulate.

|  | **Aerodynamic diameters, D_IA_** | | **Optical diameter sizes, D_IO_** | | | | |  |
| --- | --- | --- | --- | --- | --- | --- | --- | --- |
| **Quantity** | **TiO_2_** | **SiO_2_** | **TiO_2_** | **SiO_2_** | **DU U_3_O_8_** | **NatU UO_3_** | **HEU U_3_O_8_** |  |
| D_IA_ (µm) or D_IO_ (µm) | 1.54 | 2.08 | 1.82824 | 1.17278 | 1.22363 | 0.80191 | 1.5826 | Measured irregular particulate diameters |
| ρ_B_ (g/cm^3^) | 0.77 | 2.32 | 0.77 | 2.32 | 8.3 | 8.3 | 8.3 | Bulk powder density |
| ρ_0_ (g/cm^3^) | 1 | 1 | 1 | 1 | 1 | 1 | 1 | Reference density |
| χ, unitless | 1.745 | 1.36 | 1.745 | 1.36 | 1.745 | 1.745 | 1.745 | Shape factor from online source** |
| D_AE_ (µm) | 1.02 | 2.72 | 1.21 | 1.53 | 2.67 | 1.75 | 3.45 | Aerodynamic equivalent diameter |

**Mass of an individual particulate**

Because the Stokes equivalent sphere is a hypothetical particulate with the same bulk density as the loose powder, the Stokes diameter may be defined from Equation 1:

$$D_{S}= D_{AE}\sqrt{\frac{\rho_{0}}{\rho_{B}}}$$

S. Eq. (4)

The mass of an individual aerosol particulate, according to its Stokes diameter, is

$$m\left( particulate \right)= \rho_{B}\frac{\pi}{6} D_{S}^{3}$$

S. Eq. (5)

**S5 Table. Determination of the mass of the particulates.** Observed aerodynamic diameters and optical diameters were used to calculate the mass of the particulates. The units are: ρ_B_ = bulk powder density measured from the mass to the volume ratio of loose powder, D_IA_ = “diameter irregular aerodynamic” (physical size of the particulate in an aerodynamic sizer), D_IO_ = “diameter irregular optical” (physical size of the particulate in an optical sizer), D_STOKES_ = Stokes particulate diameter, m = mass of the particulate in picograms (pg).

| **Mass of an individual particulate (pg)** | | | |  |  |  |  |
| --- | --- | --- | --- | --- | --- | --- | --- |
|  | **Aerodynamic diameters, D_IA_** | | **Optical diameter sizes, D_IO_** | | | | |
| **Quantity** | **TiO_2_** | **SiO_2_** | **TiO_2_** | **SiO_2_** | **DU U_3_O_8_** | **NatU UO_3_** | **HEU U_3_O_8_** |
| D_STOKES_, µm | 1.17 | 1.78 | 1.38 | 1.01 | 0.93 | 0.61 | 1.20 |
| ρ_B_ (pg/µm^3^) | 0.77 | 2.32 | 0.77 | 2.32 | 8.3 | 8.3 | 8.3 |
| m(particulate, pg) | 0.64 | 6.89 | 1.07 | 1.24 | 3.45 | 0.97 | 7.47 |
