# Supplementary figures and images for: In vitro toxicity assessment of uranium particulates on different human lung epithelial cell models
Source: PLoS One. 2025 Oct 31;20(10):e0334247. doi: 10.1371/journal.pone.0334247 (PMC12578232; doi:10.1371/journal.pone.0334247)

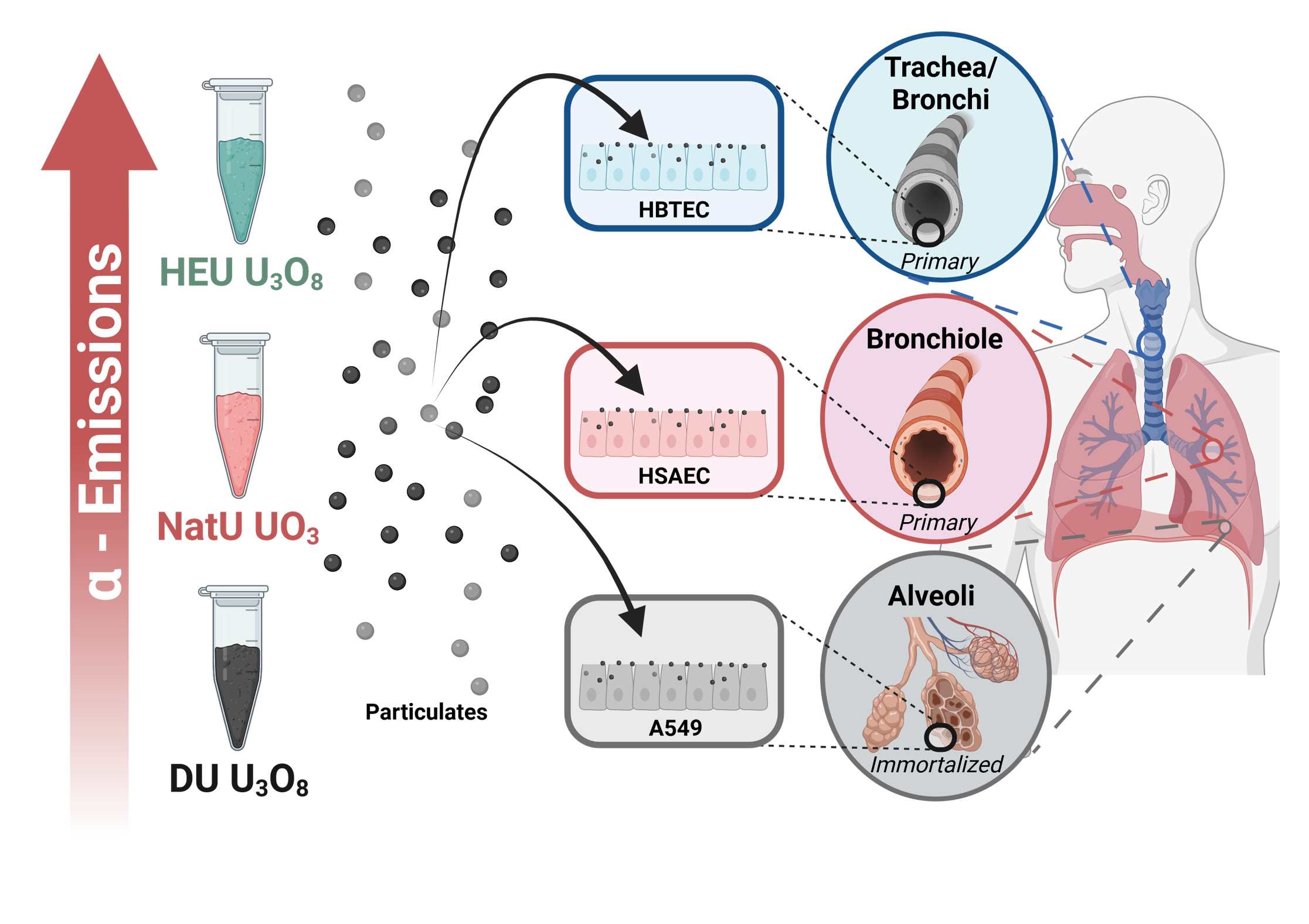

Supplement: S1 Fig — Three different enrichment states and two oxide states of uranium particulates were separately introduced to three distinct submerged in vitro human epithelial lung cell lines to investigate if increased alpha emissions, oxidation state, or respiratory tract location causes differences in toxicological effect. (TIF) [file pone.0334247.s002.tif]
